# Role of digitalization, digital competence, and parental support on performance of sports education in low-income college students

**DOI:** 10.3389/fpsyg.2022.979318

**Published:** 2022-10-20

**Authors:** Zongxi Li, Olena Slavkova, Yong Gao

**Affiliations:** ^1^School of Physical Education, Henan Institute of Science and Technology, Xinxiang, China; ^2^Faculty of Economics an Management, Sumy National Agrarian University, Sumy, Ukraine

**Keywords:** digitalization, digital competencies, sport education performance, low-income college students, parental support

## Abstract

Educational institutions have failed to achieve desired goals due to the lack of technology adoption, and this situation needs researchers’ emphasis. Hence, the current study examines the impact of digitalization and digital competencies among students on the educational performance of low-income college students in China. The article also investigates the moderating impact of parental support at the nexus of digitalization, digital competencies among students, and educational performance in low-income college students in China. The questionnaires were used by the researchers to gather the data from the selected respondents. The article has applied the smart-PLS to check the linkage among understudy constructs and test the hypotheses. The results revealed that digitalization and digital competencies among students positively link educational performance. The results also exposed that parental support significantly moderates digitalization, digital competencies, and educational performance in low-income college students in China. This article helps policymakers develop policies to improve educational performance using technology adoption.

## Introduction

Knowledge management is an asset for organizations that seek to attain a competitive position in the market. According to the knowledge-based view, organizations that possess the capability to share knowledge effectively create economic and non-economic values better than others (Grant and Phene, 2021). In the current era, digitalization has become a vehicle to extract and share knowledge in the organization. Due to its rapid growth, it is essential to find out the effects of digitalization in contemporary organizations (Colbert et al., 2016). The effect of information technology has always remained a point of concern for IT economics after realizing the fact that information technology may increase productivity in the organization (Sun, 2017). Based on work design theory, Wang et al. (2020) suggest that information technology affects the employees through three work design aspects, i.e., job demand, job autonomy, and relational aspects. Extant research shows paradoxical effects of digitalization on employees’ autonomy (Mazmanian et al., 2013; Bader and Kaiser, 2017; Gerten et al., 2019). There exists a stream of research that suggests the positive effects of technology on employees. Contrary to this, a stream of research also suggests the negative relationship between digitalization and autonomy. Therefore, there is a need for further research on the relationship between digitalization and employees’ autonomy (Wang et al., 2020).

The world is getting digitalized at every next step. It is education that makes it possible to educate the world regarding digitalization. Usually, the world focuses more on the education that pertains to business, law, and other science-related curricula. Although digitalization has expressed its involvement in education, there is a lack of involvement witnessed in the case of sports-related education. Prior to that, although the developed countries might pay focus on sports-based education in the case of developing countries, fewer evidence has been reported regarding world-class level educational facilities. China is considered the world leader almost in every field of life. The Chinese education system is digitalized from every aspect. Despite being one of the advanced countries in the world, China is also lacking results proposed for sports education. The youth of China prefer more to those studies, which allow them to earn more. Such a societal approach results in less interest in sports-related education. Sports education in China is facing less development; the few reasons for less development are as follows: (1) the educational institution’s administration usually underestimating the sports education-related courses (Popova et al., 2020), (2) the lack of sports-related courses inspection, (3) less focus on the institutions in the sports-related education development, and (4) non-availability of the sports facilities in the institutions.

**FIGURE 1:**
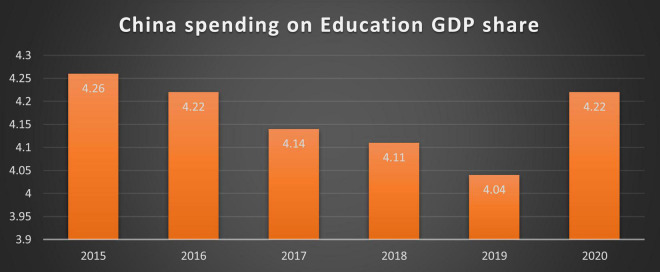
Spending on education by China.

Over the past few years, China is witnessing development in the sports education field. Around 3,000 sports schools have been created in China, with boarding schools accounting for roughly 100 of the most prominent. Approximately 300,000 students attend these institutions. There are 20 main programs and 200 small ones. Almost all of China’s Olympic athletes have come from these institutions and programs. In 2005, over 400,000 pupils were enrolled in sports schools. Approximately 6,000 professional athletes retire each year, with about 40% of them having problems finding work (Zheng et al., 2018; Chen and Chen, 2021). They are no longer cared about by the cradle-to-grave system that used to exist. When certain athletes retire, they are regularly promised jobs as police officers, but these promises are frequently violated. Although China is expressing much interest in all fields of education, there is less percentage reported in the case of sports-related education as compared to others such as business, law, and medicine. China is trying its best to become the best in almost every competition with the other countries, especially with Europe and America. University-level education in China and other developing countries uses many different strategies to improve students’ self-motivation and athletic skills. The teachers of primary, secondary, and university levels have a great role in raising the self-motivation of students related to sports and other physical activities. The student at a younger age can learn more from physical activities and motivational speeches. Also, physical fitness is traditionally considered important in Chinese culture. Currently, in China, there are many fitness clubs and fitness gyms in operation that provide physical and other sports-related education. This health and fitness-related fitness can be shown in the number of cyclists in China, which are enormous. As of 2012, there are more than 470 million bicycles in China (Dai and Menhas, 2020). Basketball is considered the most desirable game for the youth in China. They are participating in Olympic Games, along with winter and summer games from the period after 1932. For a country to be successful in sports, it has to focus on it from the school level of the students. Among many factors that influence children’s physical activities, member of the family and parents also plays an important role in promoting and shaping their children’s behaviors. Although China is producing good results in sports, there is fewer sports facilities’ development witnessed in comparison with other traditional studies such as business, law, and medicine. The Chinese spending on education is given in Figure 1.

**FIGURE 2:**
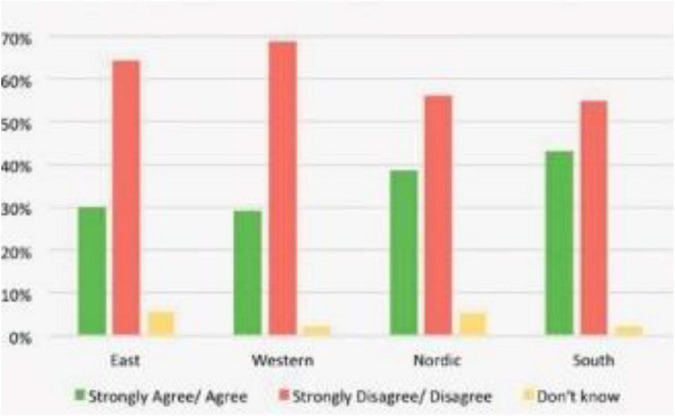
Digitalization is fancy word for new technologies.

The study examines the impact of digitalization and digital competencies among students on the educational performance of low-income college students in China. The article also investigates the moderating impact of parental support at the nexus of digitalization, digital competencies among students, and educational performance in low-income college students in China. This study has the following contribution in the literature. This study will try to address some gaps that were never identified before, such as: (1) the rapid changes in the world also having their effect on the education field. Physical education has also become more important than other forms. Consequently, being one of the important topic although researched although but still not reached its peak in case of China as there are number of its aspects are need to be explored, (2) Dugalić (2018) investigated the triangle of sports, media and digitalization, whereas this study will work on the relationship between digitalization and performance of sports education by employing the moderating factor from Chinese perspective with the fresh dataset, (3) the model consists of digitalization, digital competence, parental support, and sports educational performance which is not tested before from Chinese perspective with fresh dataset in recent time, (4) Rapoport et al. (2020) worked on physical culture digitalization at the regional level, whereas this study will check the impact of digitalization and competence on sports educational performance by employing parental support as a moderating variable in China by selecting the fresh dataset, (5) Opstoel et al. (2019) worked on the physical education development, whereas this study will check the triangle of digitalization, competence, parental support, and sports educational performance in China, and (6) Wicahyani et al. (2021) checked the effect of digital literacy in sports education, whereas this study will check digitalization and sports education relationship by employing moderating variable, i.e., parental support in China by selecting the fresh dataset (see Figure 2).

The significance of the study is as follows: (1) it will highlight the importance of digitalization for the betterment of sports education, particularly in China, (2) it will help the Chinese sports-related professionals to revamp their policies regarding the betterment of the sports education with the aim to implement the digitalization in sports education, and (3) it will also help the researchers to explore more aspects of digitalization which will affect the sports educational performance in China.

In case of structure, this article is divided into different phases. The first phase of the study presents the study introduction. In the second phase of the study, the evidence regarding digitalization, digital competence, parental support, and sports educational performance in light of the past studies is presented. The third phase of the study shines the spotlight on the methodology applied for the collection of data pertaining to digitalization, digital competence, parental support, and sports educational performance and analyzes its validity. In the fourth phase of the study, the results of the study are discussed, and later in light of other authors’ results having a similar nature, the approval of the results is discussed. The study implications, as well as conclusion, are presented. Further, this section also presents the conclusion and future recommendations for the scholars.

## Literature review

In more recent publications, definitions of digital literacy point toward cognitive skills and competences (Mishra et al., 2017). Bennett (2014) and Traxler and Lally (2016) point out that the individual is in focus as opposed to the social dimensions of learning in Beetham’s definition that highlights the cognitive perspective of digital literacy as “the functional access, skills and practices necessary to become a confident, agile adopter of a range of technologies for personal, academic and professional use” (Beetham and Sharpe, 2011; Rapoport et al., 2020; Wicahyani et al., 2021). Chan et al. (2017) also refer to cognitive skills and define digital literacy as “the ability to understand and use information in multiple formats with emphasis on critical thinking rather than information and communication technology skills.”

Educational institutions have failed to achieve desired goals due to the lack of technology adoption, and this situation needs researchers’ emphasis. Thus, the article examines the impact of digitalization and digital competencies among students on the educational performance of low-income college students in China. The article also investigates the moderating impact of parental support among the nexus of digitalization, digital competencies among students, and educational performance in low-income college students in China. Education plays a vital role in the self and professional development of people and students. There are many factors through which the educational performance could be easily enhanced. Over the past few decades, technological innovation has significantly helped the educational sector enhance its performance. This performance is not only for the development of students in the professional fields but also for the critical assessment. These assessments are differentiated in many fields of life where education is most considerably important. Sports education is vitally important for people and students. The education also varies from different fields where the students are specialized in their respective sector. Education is necessary for everyone, so it can be any type of education. Sport education is among the most important types of education. Today, the world is on the verge of innovation, and every country makes efforts to bring digitalization. In this context, Shutova and Andryushchenko (2020) checked the performance of sports education after being digitalized according to the Russian perspective. Digitalization helps Russia to overcome the challenges in sports education. Eighty-five percent of the student communities have information on the internet, and categorization tools need to be advanced to improve the grassroots initiative activity in sports education. Many universities have implemented this and show many positive outcomes. Dummy systems are developed and improve sports educational performance. The study concluded that digitalization in ongoing university sports education facilitates it in both a theoretical and practical manner.

Sports education contains many types of education. Some are the learning skills of certain sports. In this context, Erawan et al. (2020) checked the relation between digitalization in wrestling sports and its performance. Wrestling needs to master the basic technique; for this learning, Joint Lock is the first step of learning. Digitalizing this basic technique is very innovative in the learning process. This learning aims to strengthen students’ cognitive approach and psychomotor abilities. This digitalization improves the four basic movements of wrestlers. The results have been observed that before digitalization, many wrestlers suffered injuries due to misunderstandings in lectures; now, digitalization opens many ways of learning. The conclusion of all is that the emergence of the digital era in sports education has become a source of knowledge for students. They can also learn directly from the internet. Digitalization covers all aspects of life, especially sports. The emphasis is on diversity in its implementation. Sports much need digitalization because sports are broadcast to a huge audience with the help of a digital medium. In this context, Dugalić (2018) checked the relation between digitalization and sports. Digitalization in sports opens numerous research approaches. When digital marketing has come with digital journalism, they create controversies, but these are in small numbers. Digitalization not only helps celebrity in the perspective of marketing but also helps athletes who can play back their performance on video and learn from their mistakes. Digital marketing also helps sports in terms of attracting audiences and financers. Siberian University computed the result that the main source and a huge source of earning for sports is digital marketing, almost 72% of total income. It is concluded that digitalization in sports opens numerous paths regarding legislation ethics, a field of implementation, and the perspective of future development of sports education. Thus, the hypothesis derived from the above debate is as follows:

**H1:** The digitalization has positive influences on the sports educational performance.

In educational performance, the role of technological advancement is most important. Technology has supported education in various ways. These ways have enabled people and students to attain relevant education from their institutions. It is possible with the technology that has induced significant feasibilities in the education sector. More importantly, the competence structure in the education sector also inserts its dominant role. This role is widely supported by the induction of technological innovation. The role of technology has enabled the students to attain education with the help of digital technology and its competence. The facts and explanations elaborated by the digital competence structure have increased the knowledge among students that increased educational performance. Physical or sports education has a huge spot for significant making and a center site for value-based teachings. In this context, Engen and Engen (2019) examined the relationship between sports educational performance and digital competence. Physical education is identified as a critical setting in which to look at a percentage of the challenges shown by understudies and teachers as to values-based rehearses in carefully mediated spaces. In Norway, digitalization in the educational sector is a major concern for governments. In the early days of digitalization, much effort was spent on enhancing the competencies of teachers digitally, and it had great effects on the sports performances of their pupils. The approach of domestication of technology is used for research purposes. Data were gathered from research in the time frame of 2011–2014 and from the early digitalization era of the 1990s. In conclusion, the term teachers’ expert advanced ability is connected to circumstances and substantial purposes, and it is accordingly, as of now, not achievable to discuss only one skill but instead of a few between associated capabilities. This will relatively groom the skill set of their pupils. Flipped learning is new digital innovation in physical education applied with the help of information and communication technology (ICT). Flipped learning emerges new methodologies in physical education or sports education. In this context, Hinojo Lucena et al. (2020) examined the relationship between digital competence and sports education. The study aimed to investigate the changes in the performances of physical education students with and without flipped learning simultaneously. In Spain, 119 students participated in a real-life experiment for one institute. The students were divided into two groups; for the one group teachers were not digitally competent with flipped learning techniques and flipped learning techniques were used for the other to identify changes in the performance. The results showed that the group with flipped learning was more motivated and autonomous than other groups. The results proposed that traditional learning has some shortcomings, and the digital incompetence of teachers might be a reason for the better performance of physical education of pupils required to introduce new methods like flipped learning. Thus, the hypothesis derived from the above debate is as follows:

**H2:** The digital competence has positive influences on sports educational performance.

Eventually, parental involvement is also considered vital for the enhancement of sports educational performance. It is somehow necessary due to the physical as well as financial support that is required by the students in their fields. The structure of the family is also important among the students that are attaining sports education. There is a probability of individual efforts too in sports education, but the element of inheritance could not be eliminated. Most of the students in sports education are successful due to their blood belonging to their forefathers. This element could not be neglected, and when supported by technological advancement, there is significant nourishment among students. As the importance of parental support is vital for the betterment of the student’s performance, in this context, Rhodes et al. (2020) investigated the involvement of parental support in youth activities. The results of the study revealed that parental support is the key to the analysis and betterment of the youth activities, particularly education. Although the sports-related education is rated important in developed countries, in the case of developing countries, there is a need to promote it. The students all around the globe choose less sports education in comparison with other education like a business, and information technology. Here, parental support motivates the students who select sports education. Subsequently, Moral-García et al. (2020) also checked the parental support relationship with students’ academic performance, whether it is sports or non-sports. The results of the study revealed that parental support played a vital role in the betterment of the educational performance of the students. The student’s sports-related activities are very important for their focus on sports-related education. The physical activities decided by the parents for the students develop their interest toward the education which student will choose for the future. In this context, Reimers et al. (2019) investigated the effect of parental support on the student’s physical activities. The results of the study revealed that parental and peer supports play a vital role in the choice and betterment of the student’s educational performance. Education is critical for people’s and students’ personal and professional development. There are numerous factors that can be used to improve educational performance. Over the last few decades, technological advancements have greatly aided the educational sector in improving its performance. As the world is getting digitalized, this digitalization effect can also be seen in every field, particularly education. Education is the field that will ensure the proper understanding and implementation of digitalization in every field. Although digitalization is usually witnessed in other fields of life, there is less focus witnessed in sports-related education. Parental support plays a vital role in sports educational performance. In this context, Haroona et al. (2018) investigated the moderating role of parental support in sports participation and academic achievement. The results revealed that parental support significantly moderates the relationship and encourages the students to participate in sports-related educational activities with the aim of producing academic achievement. Thus, the hypothesis derived from the above debate is as follows:

**H3:** Parental support positively moderates digitalization and sports educational performance.

The role of a parent is considered a key factor in the educational experience of youth sports. Usually, the parents are responsible for the introduction of sports and physical education among the students and children. Their involvement in student education is deemed a requirement for students and children upgrading from the early stages. The involvement of digital competence among the parents and sports educational performance is also important. The introduction of digital competence has significantly influenced sports educational performance. It has a moderating role of parental support through digital competence for enhancing the educational performance of sports. With parental support, the digital competence structure in the educational sector has uplifted the values of students and enhanced their performance. In this context, Spante et al. (2018) conducted a systematic review on the relationship between digital competency and education. Digitalization promotes the literacy rate as, in recent times, better and easy forms of education are introduced which are accessible. The accessibility helps the parents and the students to get their children’s educated. Thus, digitalization ensures the betterment of education literacy. Here, parental support plays a vital role. In this context, Ede et al. (2022) investigated the parent’s engagement in students’ achievement of students’ education. The results of the study revealed that parental support in any form plays a vital role in students’ achievement of desired education level. The proper parental guidance and support help their children to achieve their desired education. The students who lack parental support usually fail to achieve the desired education. The importance of technology innovation in educational success cannot be overstated. Technology has aided education in a variety of ways. People and students have been able to obtain appropriate education from their universities thanks to these methods. It is possible thanks to technological advancements that have opened up new possibilities in the field of education. Subsequently, Hu and Wu (2020) investigated the role of parents’ support in education. The results of the study revealed that parental support plays a vital role in terms of the achievement of students’ desired education. Further, Boonk et al. (2018) also investigated the parent’s role of the academic achievement of the students. The results proposed that the children who get support by their parents result in producing better educational results. Parental support acts as a motivator for the children to achieve their desired education. Thus, the hypothesis derived from the above debate is as follows:

**H4:** Parental support significantly moderates digital competence and sports educational performance.

The above studies have the following gaps which are tried to incorporate in the given research. Physical education has also become more important than other forms. Consequently, being one of the important topic although researched although but still not reached its peak in case of China as a number of its aspects need to be explored. Dugalić (2018) investigated the triangle of sports, media, and digitalization, whereas this study will work on the relationship between digitalization and performance of sports education by employing the moderating factor from Chinese perspective with the fresh dataset. The model consisting of digitalization, digital competence, parental support, and sports educational performance is not tested before from Chinese perspective with fresh dataset in recent time. Opstoel et al. (2019) worked on the physical education development, whereas this study will check the triangle of digitalization, competence, parental support, and sports educational performance in China. Wicahyani et al. (2021) checked the effect of digital literacy in sports education, whereas this study will check digitalization and sports education relationship by employing moderating variable, i.e., parental support in China by selecting the fresh dataset.

## Research methodology

### Data overview

The study examines the impact of digitalization and digital competencies among students on educational performance and also investigates the moderating impact of parental support among the nexus of digitalization, digital competencies among students, and educational performance in low-income college students in China. The questionnaires were used by the researchers to gather the data from the selected respondents. The measurement scale for the variables was taken from past studies. The measurement scale for digitalization consists of four items taken from Zeshan et al. (2021), while the measurement scale for digital competencies consists of five items taken from Barragán-Sánchez et al. (2020). The measurement scale for parental support consists of four items extracted from Wilk et al. (2018), while the measurement scale for educational performance consists of four items taken from Mehrvarz et al. (2021). Table 1 shows the measurement scale of the variables.

**TABLE 1:** Measurement of the constructs.

Items	Questions	Sources
**Digitalization**		
DG1	“I use the information system a lot to find the solutions for the education system.”	Zeshan et al., 2021
DG2	“I depend heavily on the information system to fulfill my responsibilities.”	
DG3	“My company’s information system is essential to my work.”	
DG4	“Without an information system, it would be very difficult to do my job.”	
**Digital competence**		
DC1	“I am clear about the technologies’ impact on my education.”	Barragán-Sánchez et al., 2020
DC2	“I have heard about the education improvement by using technologies.”	
DC3	“You could clearly define the technologies impact on the educational performance.”	
DC4	“I have received courses on the use of technologies in education.”	
DC5	“I would know how to define the technological uses in the educational system perfectly.	
**Parental support**		
PS1	“Encouraged children to be physically active and using the technology.”	Wilk et al., 2018
PS2	“Provided facilities to be active desired goals in their education.”	
PS3	“Watched children participate in technology usage programs in their educations.”	
PS4	“Active with children in learning technologies to support their education.”	
**Educational performance**		
EP1	“I am confident about the adequacy of my academic skills and abilities.”	Mehrvarz et al., 2021
EP2	“I feel competent conducting my course assignments.”	
EP3	“I have learned how to perform my coursework efficiently and successfully.”	
EP4	“I have performed academically as well as I anticipated I would.”	

The students of low-income colleges working in China are the respondents of the study. The respondents were selected using simple random sampling. The survey questionnaires were sent to the respondents by personal visits to the colleges. The researchers have sent around 561 surveys, but only 290 were received after two weeks and represent approximately 51.69% response rate.

### Estimation techniques

The article has applied the smart-PLS to check the linkage among understudy constructs and test the hypotheses. It is an appropriate tool for the analysis of primary data due to its feature of dealing with large and small datasets (Ringle et al., 2015). The PLS-SEM is used when the analysis is concerned with testing a theoretical framework from a prediction perspective. When the structural model is complex and includes many constructs, indicators, and/or model relationships, our research has a path model that includes one or more formatively measured constructs.

There is no doubt that PLS-SEM has become very popular for survey research in recent years since its introduction in 1966 by Herman Wold. The development of PLS-SEM is largely driven by its advantages in distributional assumptions, the absence of factor indeterminacy, and models with more parameters than observations (Dijkstra and Henseler, 2015a). The PLS-SEM is regarded as a variance-based approach to SEM (Chin et al., 2003; Tenenhaus, 2008) which becomes appreciated for its ability to estimate both composites and factors (Henseler et al., 2016). Dijkstra and Henseler (2015b) support that PLS-SEM has “the possibility of estimating models having more variables or parameters than Observations.” Although few studies in CBSEM focused on developing a large model using small sample size, these models are restricted by three items per LV to achieve goodness of fit (e.g., Marsh et al., 2004; Barendse et al., 2010).

**FIGURE 3:**
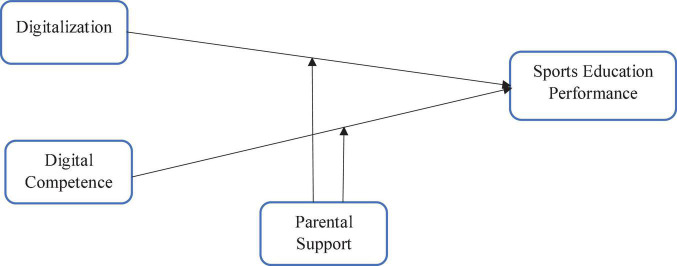
Theoretical model.

The study has used two independent variables, namely, digitalization (DG) and digital competencies (DC), while the study has used one moderating variable, namely, parental support (PS), and also used one dependent variable, namely, sports educational performance (EP). These variables are presented in the framework given in Figure 3. The moderating variable parental support affects the sports in educational performance with digitalization and digital competence.

### Research findings

The results examined the convergent validity of item correlation with the help of average variance extracted (AVE) and factor loadings. The results exposed that the values of both the measures are larger than 0.50. The results exposed valid convergent validity. In addition, the results also examined the reliability with the help of composite reliability (CR) and alpha. The results exposed that the values of both the measures are higher than 0.70. The results exposed valid reliability. Table 2 shows these outcomes.

**TABLE 2:** Convergent validity.

Relationships	Beta	Loadings	Alpha	CR	AVE
Digital competence	DC1	0.930	0.945	0.958	0.820
	DC2	0.866			
	DC3	0.933			
	DC4	0.865			
	DC5	0.931			
Digitalization	DG1	0.917	0.951	0.965	0.872
	DG2	0.918			
	DG3	0.946			
	DG4	0.953			
Educational performance	EP1	0.635	0.843	0.897	0.689
	EP2	0.885			
	EP3	0.897			
	EP4	0.876			
Parental support	PS1	0.882	0.897	0.928	0.764
	PS2	0.874			
	PS3	0.854			
	PS4	0.886			

In addition, the results also examined the discriminant validity with the help of cross-loadings and Fornell–Larcker. The results revealed that the values that indicated the linkage with construct itself are higher than those that exposed the linkage with other constructs. The results exposed valid discriminant validity. Tables 3, 4 show these outcomes.

**TABLE 3:** Fornell–Larcker.

	DC	DG	EP	PS
DC	0.906			
DG	0.501	0.934		
EP	0.438	0.383	0.830	
PS	0.397	0.409	0.347	0.874

**TABLE 4:** Cross-loadings.

	DC	DG	EP	PS
DC1	**0.930**	0.460	0.390	0.337
DC2	**0.866**	0.441	0.414	0.386
DC3	**0.933**	0.463	0.382	0.341
DC4	**0.865**	0.442	0.406	0.389
DC5	**0.931**	0.459	0.383	0.338
DG1	0.460	**0.917**	0.358	0.410
DG2	0.467	**0.918**	0.377	0.363
DG3	0.467	**0.946**	0.344	0.382
DG4	0.475	**0.953**	0.349	0.373
EP1	0.243	0.333	**0.635**	0.226
EP2	0.401	0.309	**0.885**	0.288
EP3	0.405	0.331	**0.897**	0.337
EP4	0.382	0.308	**0.876**	0.291
PS1	0.349	0.344	0.298	**0.882**
PS2	0.312	0.341	0.312	**0.874**
PS3	0.363	0.376	0.276	**0.854**
PS4	0.365	0.371	0.325	**0.886**

The bold values indicate the results for the corresponding statistics for the whole variable, not the items.

Moreover, the results also examined the discriminant validity with the help of the heterotrait–monotrait (HTMT) ratio. The results exposed that the values are lower than 0.90. The results exposed valid discriminant validity. Table 5 shows these outcomes.

**TABLE 5:** Heterotrait–monotrait ratio.

	DC	DG	EP	PS
DC				
DG	0.528			
EP	0.485	0.433		
PS	0.430	0.443	0.398	

The results shown by path analysis revealed that digitalization and digital competencies among students have a positive linkage with sports educational performance and accept H1 and H2. In addition, the findings exposed that with the 1% increase in digital competencies, the sports educational performance will also increase by 0.307% and vice versa. Additionally, the findings also exposed that with the 1% increase in digitalization, the sports educational performance will also increase by 0.181% and vice versa. Moreover, the results also exposed that parental support significantly moderates among digitalization, digital competencies, and sports educational performance in low-income college students in China and accepts H3 and H4. These associations are given in Table 6.

**TABLE 6:** Path analysis.

Relationships	Beta	SD	*T*-statistics	*P*-values
DC → EP	0.307	0.072	4.235	0.000
DC × PS → EP	0.102	0.059	1.732	0.042
DG → EP	0.181	0.081	2.237	0.013
DG × PS → EP	0.129	0.069	1.881	0.030
PS → EP	0.215	0.064	3.353	0.000

## Discussion of the results

**FIGURE 4:**
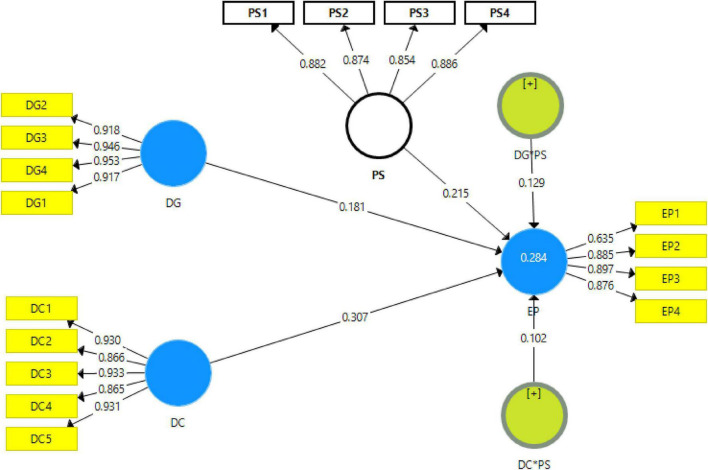
Measurement model assessment.

**FIGURE 5:**
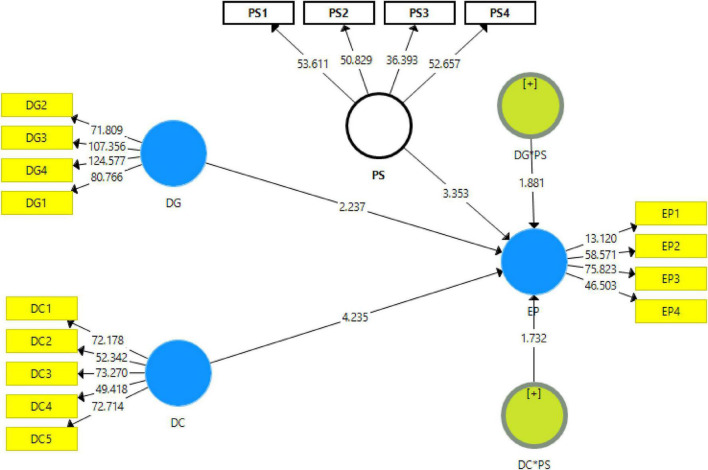
Structural model assessment.

The article examines the impact of digitalization and digital competencies among students on the educational performance of low-income college students in China. The article also investigates the moderating impact of parental support among the nexus of digitalization, digital competencies among students, and educational performance in low-income college students in China. The results indicated that digitalization has a positive impact on sports educational performance. These results are supported by Shutova and Andryushchenko (2020), which examines the role of digitalization in sports educational performance. The study highlights that many educational institutions are providing sports education to students. The success of sports education depends on the ways which the coach or leaders adopt to provide sports education or training. When they digitalize their methods to educate sports students, they can provide enhanced sports-related knowledge to students as digitalization increases the source of information. These results are also in line with Soltani and Morice (2020), which states that digitalization allows the coach to utilize innovative digital technologies such as sensors, wearable sports tech, reflection, and digital protective equipment, which assists in checking the mental and physical performance of students during training, since the weaknesses, highlights these weaknesses on the students, and brings improvement in their athletic performance. Hence, digitalization improves sports educational performance. These results are also in line with Mutz et al. (2021), which states that the use of digital technologies like high-end wearable technological products is helpful to measure the metrics such as speed, time, accuracy, and frequency of athletes’ physical movements, track sleep, perform heart monitoring and ECG functionalities, as well as give instructions how the mental and physical fitness of athletes can be improved. Hence, the digitalization of training processes improves sports educational performance (see Figures 4, 5).

**FIGURE 6:**
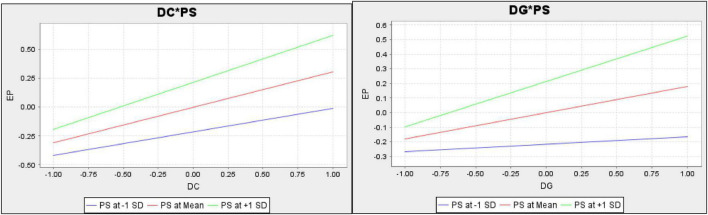
Moderation analysis.

The results revealed that parental support is a suitable mediator between digitalization and sports educational performance. These results are also supported by Hertling et al. (2022), which states that the process of digitalization of training student–athletes requires large financial resources. If parents of students provide financial support and make contributions on their own individual part, the professional sports education team can introduce innovative technologies specially designed to help sports training. Moreover, when parents adopt supportive behavior toward the children while they are getting sports training, it helps them learn well. So, in case the parents are supportive, it is easy for the tutors or trainers to digitalize training processes and effectively perform in providing sports education. These results are supported by Shaalan and Abd Ali (2021), which shows that when parents are attentive toward the children while they are getting training education in specific sports and adopt a supportive behavior, they not only rely on the sports education from the institution but themselves try to help children learn through digital technologies. In this situation, the professional sports trainers can better implement digitalization, effectively educate the students, and can have better results from a student during tests and matches. Hence, parental support improves digitalization and sports educational performance and the contribution of digitalization to sports educational performance (see Figure 6).

The results indicated that digital competencies have a positive impact on sports educational performance. These results are supported by Hava (2021), which analyzes the role of digital competencies in sports educational performance. This study reveals that if the digital professionals have digital competencies like knowledge of digital technologies to be used in general and in sports and the cognitive and physical skills to interact with and utilize them, they can better perform as tutors or coaches in sports training programs. Having digital competencies, they can better watch players’ actions, assess their performance and their sports psychological and physical development, and help them do a better rehearsal. Hence, digital competencies development improves sports educational performance. These results are also in line with those of Yaman (2021), which highlights that the educational institution, which provides the facility of providing sports education and the attention toward the digital competencies in sports trainers and helps create a better sports training environment, enhance students’ sports knowledge, improve the mental and physical fitness of players, and improve the skills required for a specific game. So, the sports educational performance of the institution is improved. These results also match with those of Ahir et al. (2020), which shows that the digital competencies such as knowledge, skills, and attitudes to make creative, confident, and critical use of digital technology and systems enable the student–athletes to have a more informative, cooperative, and persuasive sports learning environment. So, sports educational performance is improved.

The results indicated that parental support is an appropriate moderator between digital competencies and sports educational performance. These results are supported by Qurban et al. (2019), which highlights that parents play a key role in students’ education. The parents have the aptitude that they must emotionally and financially support their children in getting sports education; they keep on sports technological advancements and assist them in learning the digital competencies to benefit from these technologies. When students in sports training classes already have digital competencies, it becomes easy for the training staff to apply digital technologies in the training process and train them efficiently. Hence, parental support improves digital competencies and sports educational performance and strengthens their relationship. These results are also in line with those of Moral-García et al. (2020), which reveals that when parents of children who want to proceed in the sports field and intend to have professional training are supportive, they try their best to find an efficient sports training center that is operating its functions on a digital basis. And when the parents provide support to children in rehearsal, they can better get a sports education. That is why parents’ support builds and improves the link between digital competencies and sports educational performance. These results also match with those of Cheng et al. (2022), which shows that the support from parents assists students in applying digital technologies and performing well in getting a sports education.

## Conclusion

The objective of this study was to examine the impacts of digitalization and digital competencies on sports educational performance. One of its objectives is also to analyze the role of parental support between digitalization and digital competencies and sports educational performance. The authors followed a questionnaire distributing research method and acquired quantitative empirical information for digitalization, digital competencies, parental support, and sports educational performance from China. This empirical information and its analysis showed a positive relation between digitalization and digital competencies to sports educational performance. The results indicated that the success of sports education depends on the ways adopted to provide sports education or training. When these methods are digitalized, the tutors have better information ability to sense the students’ game performance and develop skills, and thus, they can better educate them in the sports field. The results showed that digital competencies such as knowledge, abilities, and attitudes that enable student–athletes to make creative, confident, and critical use of digital technology and systems provide for a more informative, cooperative, and compelling sports learning environment. As a result, the performance of sports education has improved. The results suggest that parental support helps in digitalization and improves digital competencies and sports educational performance. So, digitalization and digital competencies’ contribution to sports educational performance increases.

The current study carries both theoretical and empirical implications. The study has a significant contribution to the literature. This study examines the impacts of digitalization and digital competencies on sports educational performance. Usually, in the previously conducted literature, the digitalization and digital competencies have been analyzed under one head, and digital technology adoption and its contribution to the sports educational performance have been analyzed. This study, which examines digitalization and digital competencies individually for sports educational performance, is an exception. This initiative to examine the parental support as a moderator between digitalization, digital competencies, and sports educational performance adds to the literature. Similarly, the analysis of digitalization and digital competencies’ impacts on the sports educational performance in China is also one of the initial attempts. The current study has great significance in the countries where one or more games are preferred to be played at the national or international level for many social, cultural, political, and economic benefits. This study highlights the ways how to improve sports educational performance. This article helps policymakers develop policies to improve educational performance using technology adoption. This study reveals that the policymakers must try to force the institution which is engaged in arranging sports education classes that they must initiate to digitalize the methods applied for students’ sports training and develop digital competencies in students as well trainers so that sports educational performance can be improved. Moreover, parents must be motivated to provide support to their children if improved sports educational performance is required.

The study has many limitations besides its great theoretical and empirical significance. These limitations are expected to be filled in future literature. First, this study throws light on only three factors, namely, digitalization, digital competencies, and parental support, for analyzing their contribution to the sports educational performance. There are many other factors, such as innovation adoption, institution support, and leadership, that have much influence on sports educational performance. In this article, these factors are missing in the analysis of sports educational performance. So, the scope of the study is limited. To make the theme of the study comprehensive, the future authors must investigate the relationship of these factors with sports educational performance as well. In this study, only a moderator like parental support between digitalization, digital competencies, and sports educational performance has been analyzed. It is recommended that future scholars must discuss at least one mediator between digitalization and digital competencies and sports educational performance.

## Data availability statement

The original contributions presented in this study are included in the article/supplementary material, further inquiries can be directed to the corresponding authors.

## Author contributions

All authors listed have made a substantial, direct, and intellectual contribution to the work, and approved it for publication.
